# Solar Wind Magnetosphere Ionosphere Link Explorer (SMILE): Science and Mission Overview

**DOI:** 10.1007/s11214-024-01126-6

**Published:** 2025-01-27

**Authors:** Chi Wang, Graziella Branduardi-Raymont, C. Philippe Escoubet, Colin Forsyth

**Affiliations:** 1 NSSC/CAS, National Space Science Center, Chinese Academy of Sciences, No.1, Nan Er Tiao ZhongGuanCun, PO Box 8701, Beijing, 100080 China; 2 Dept. of Space and Climate Physics, MSSL/UCL, Mullard Space Science Laboratory, University College London, Holmbury St. Mary, Dorking, Surrey RH5 6NT UK; 3 ESA/ESTEC (SCI-EP), Postbus 299, Keplerlaan, 1, Noordwijk, 2200 AG The Netherlands

## Abstract

The Solar wind Magnetosphere Ionosphere Link Explorer (SMILE) was proposed to the Chinese Academy of Science (CAS) and the European Space Agency (ESA) following a joint call for science missions issued in January 2015. SMILE was proposed by a team of European and Chinese scientists, led by two mission Co-PIs, one from China and one from Europe. SMILE was selected in June 2015, and its budget adopted by the Chinese Academy of Sciences in November 2016 and the ESA Science Programme Committee in March 2019, respectively. SMILE will investigate the connection between the Sun and the Earth using a new technique that will image the magnetopause and polar cusps: the key regions where the solar wind impinges on Earth’s magnetic field. Simultaneously, SMILE will image the auroras borealis in an ultraviolet waveband, providing long-duration continuous observations of the northern polar regions. In addition, the ion and magnetic field characteristics of the magnetospheric lobes, magnetosheath and solar wind will be measured by the in-situ instrument package. Here, we present the science goals, instruments and planned orbit. In addition the Working Groups that are supporting the preparation of the mission and the coordination with other magnetospheric missions are described.

## Introduction

The first China-ESA collaboration on a science mission started with the ESA/NASA Cluster mission. Following a call to build Cluster data centres in the early 1990s, Prof. Zhenxing Liu from the Center for Space Science and Applied Research (CSSAR), now called National Space Science Center (NSSC), proposed to establish a Cluster data centre in Beijing. The ESA Director of Science was surprised and interested by the proposal and decided to send a delegation made of ESA representatives and Cluster PIs to discuss further the collaboration. It was subsequently agreed to establish the Cluster Chinese data centre, under the condition that a scientific collaboration be established between the Cluster community and CSSAR within the framework Cooperation Agreement made between ESA and the People’s Republic of China signed in 1980.

A few Chinese students completed PhDs and post-docs in Cluster PI teams (MPS, Germany and CESR – now IRAP – in Toulouse, France) and at ESA establishements (ESTEC, ESRIN) to learn about Cluster and its instruments. A cooperation agreement between ESA and CSSAR on Cluster collaboration was signed on 25 November 1993 and the Chinese data centre was established in Beijing. This started a very strong collaboration between China and Europe which is shown in the high number of Chinese lead Cluster referred papers that is currently above 500 (16% of total Cluster refereed papers).

After the first Cluster launch in 1996, which failed on Ariane 5’s maiden flight, it was decided to fully rebuild Cluster and relaunch the mission. During the rebuild, Prof. Liu proposed a further collaboration on the complementary Double Star mission, consisting of two spacecraft supporting eight European instruments, including seven flight spares from Cluster II, and eight Chinese instruments. The ESA-Chinese National Space Administration agreement to develop the Double Star programe was signed on 9 July 2001. The two spacecraft were launched on two Chinese rockets in 2003 and 2004, following the successful launch of Cluster II in 2000. Running until 2008, the Cluster-Double Star collaboration provided the first 6-point plasma measurements within the magnetosphere from a suite of identical instruments and was awarded the International Academy of Astronautics (IAA) Team Achievement Award in 2010.

Following the end of the Double Star mission, ESA and CAS investigated further collaboration in solar-terrestrial science with the Kuafu programme. Unfortunately, ESA could not provide funding and the programme was stopped, however, soon after, ESA and CAS decided to collaborate on a joint science mission call. Unlike previous collaborative programs that were led by one agency, the new programme would be built on a 50/50 share between ESA and CAS. A first workshop was organized by CAS and ESA in Chengdu (China) on 25-26 February 2014, inviting both the Chinese and European science community to present science ideas for cooperative missions and give technical and programmatic constraints on possible future missions. About 50 oral presentations and posters were given by CAS and European science communities and combined teams started forming on various science goals. Six months later a second workshop was organized in Copenhagen (Denmark) on 23-24 September 2014, where the joint European and Chinese science teams presented proposed science objectives and associated early mission concepts. Nineteen missions were presented during the workshop and further technical and programmatic aspects were given by ESA and CAS.

A few months later, on 19 January 2015, the joint call for science mission proposals was issued by CAS and ESA. Thirteen proposals were received among which was the SMILE mission lead by Prof. Graziella Branduardi-Raymont and Prof. Chi Wang. A joint peer review process was conducted by scientists from the Chinese and European science community and recommended SMILE to be selected. On 4 June 2015 the mission was selected by CAS and ESA to enter Phase A. The Chinese component was officially approved by CAS in November 2016. After a few years studying in more details the mission, the SMILE definition study report was published in December 2018 (Branduardi-Raymont et al. 2018) and the mission was finally adopted by ESA on 5 March 2019.

The mission is now in its implementation phase with the production of various models of spacecraft and instruments (Li et al. 2024, this collection). The launch is currently planned in 2025 on a VEGA-C launcher from Kourou, French Guiana.

## Scientific Objectives

The Sun has a major influence on the Earth’s environment. Most of the impinging energy is in the form of electromagnetic radiation, mainly infrared, visible and ultraviolet light. A small fraction is however sent in streams of charged particles, mainly protons, electrons and He^++^, known as the solar wind, that carry with them the interplanetary magnetic field (IMF). Although the solar wind is not very dense, typically a few particles per cubic centimeter, it is the key driver of the conditions in near-Earth space. A major effect on Earth’s magnetic field is to change its dipole-like shape into a wind-sock shape by compressing the magnetic field on the Sun side and stretching into a very long tail (millions of kilometers) on the opposite side, inducing large-scale electric currents that couple distant regions of geospace into the Earth’s upper atmosphere

Although the effects of the Sun on the Earth have been studied for many 10s of years, the Sun-Earth system is so large that it is difficult to envision the overall interaction accurately from in-situ point measurements. It is therefore difficult to predict whether changes on the Sun and in the solar wind will have a small or large impact in the Earth’s near-space environment. SMILE will make new measurements that will image the place where the solar wind first encounters the Earth magnetic field, the magnetopause. Simultaneously, SMILE will provide the longest continuous intervals of images of the full northern hemisphere auroral oval made to date. To complement these measurements, the solar wind and magnetic field plasma encountering the Earth will be measured with very high precision on-board SMILE.

The interaction between Earth’s magnetic field and the incident IMF can result in reconnection between the two magnetic field regimes on the dayside and the addition of magnetic energy into the magnetosphere. The opened magnetospheric magnetic field is eventually closed in the magnetotail and convected back to the dayside magnetosphere to cycle through again (Dungey 1961). However, this process is not steady, resulting in energy build up and explosive energy release that results in rapid and widespread auroral brightenings known as substorms (e.g., Akasofu 1964; Hones 1979). The factors controlling the substorm cycle have long been debated (e.g. Angelopoulos et al. 2008; Lui 2009; Lyons et al. 1997; Freeman and Morley 2004) but remain unresolved.

Sporadically and unpredictably, the Sun ejects immense clouds of plasma and magnetic field, known as coronal mass ejections (CME), into the heliosphere. These CMEs can reach speeds in excess of 4-5 times of the ambient solar wind, driving supersonic shocks and accelerating charged particles to a significant fraction of the speed of light. Should one or more of these CMEs impact on Earth’s magnetic field, enhanced solar wind plasma coupling with the magnetosphere and ionosphere can result in geomagnetic storms in which the ring current strength increases and bursts of large-scale auroral activity last longer than normal, with the auroral oval expanding further equatorward. The arrival of CMEs can also wipe out energetic electrons in Earth’s inner magnetosphere (the radiation belts) by pushing the magnetopause sufficiently close to directly intersect the belts, before causing the conditions for rapid acceleration of electrons to replace, or often more than replace, those that were lost (e.g. Baker et al. 2019; Murphy et al. 2018). Substorms can also enhance radiation belt electron fluxes (e.g. Forsyth et al. 2016), but the link between storms, substorms and storm-time substorms is not fully understood.

As well as being of great scientific interest, Sun-Earth interactions can have direct impacts on technology in space and on the ground, with the potential to dramatically affect our daily life. These impacts include disrupting the communications, causing anomalies and decreasing the accuracy on GPS satellites, loss of low-altitude communication satellites (see e.g. Starlink accident, Fang et al. 2022), or disruption of power lines. Extreme storms, although very rare with a recurrence period of 1 in 100 to 200 years, could also have dramatic consequences on our life on Earth (report from UK Royal Academy of engineering in 2013) with severe economic consequences (Eastwood et al. 2017). Predicting solar storms and their impacts is therefore a key aim for scientists and engineers. Being able to constrain the location and dynamics of the outer boundary of the magnetosphere and the energy input into the upper atmosphere through auroral particle precipitation during such events will be a critical component to being able to better predict their future impact.

The three main science questions that the SMILE mission will address are:

1. What are the fundamental modes of the dayside solar wind/magnetosphere interaction?
2. What defines the substorm cycle?
3. How do CME-driven storms arise and what is their relationship to substorms?

We will describe below each science question as well as the measurements required to answer them.

### What Are the Fundamental Modes of the Dayside Solar Wind/Magnetosphere Interaction?

**Fig. 1:**
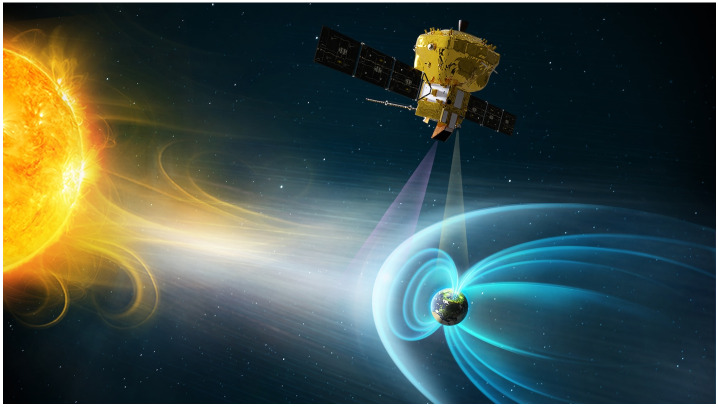
Sketch of the Sun-Earth interaction. The Earth’s magnetic field is shown with the blue lines around the Earth. In front of the magnetosphere we can see the magnetosheath in white and the solar wind in grey. The magnetosheath is bounded by the bow shock on the sun side and the magnetopause on the Earth side. SMILE instrument SXI and UVI field-of-views are shown with purple and yellow triangles respectively

The solar wind is a stream of charged particles, mainly protons, electrons and alpha particles (He^++^). In addition the solar wind contains a very small fraction of highly ionized ions such as C^5+^, C^6+^, O^6+^, O^7+^, O^8+^ and Fe^7+-17+^. The solar wind carries the interplanetary magnetic field (IMF) which, together with the above particles, impinge on the Earth magnetic field at supersonic speed (Fig. 1). The Earth magnetosphere is an obstacle for the solar wind and the interaction creates a bow shock upstream of the Earth, where the solar wind particles are decelerated and heated. Further downstream, the magnetopause is the boundary separating the solar wind that crossed the bow shock from the magnetosphere. In between the bow shock and the magnetopause lies the magnetosheath where the solar wind plasma is conpressed. This region is the focus of the SMILE mission.

**Fig. 2:**
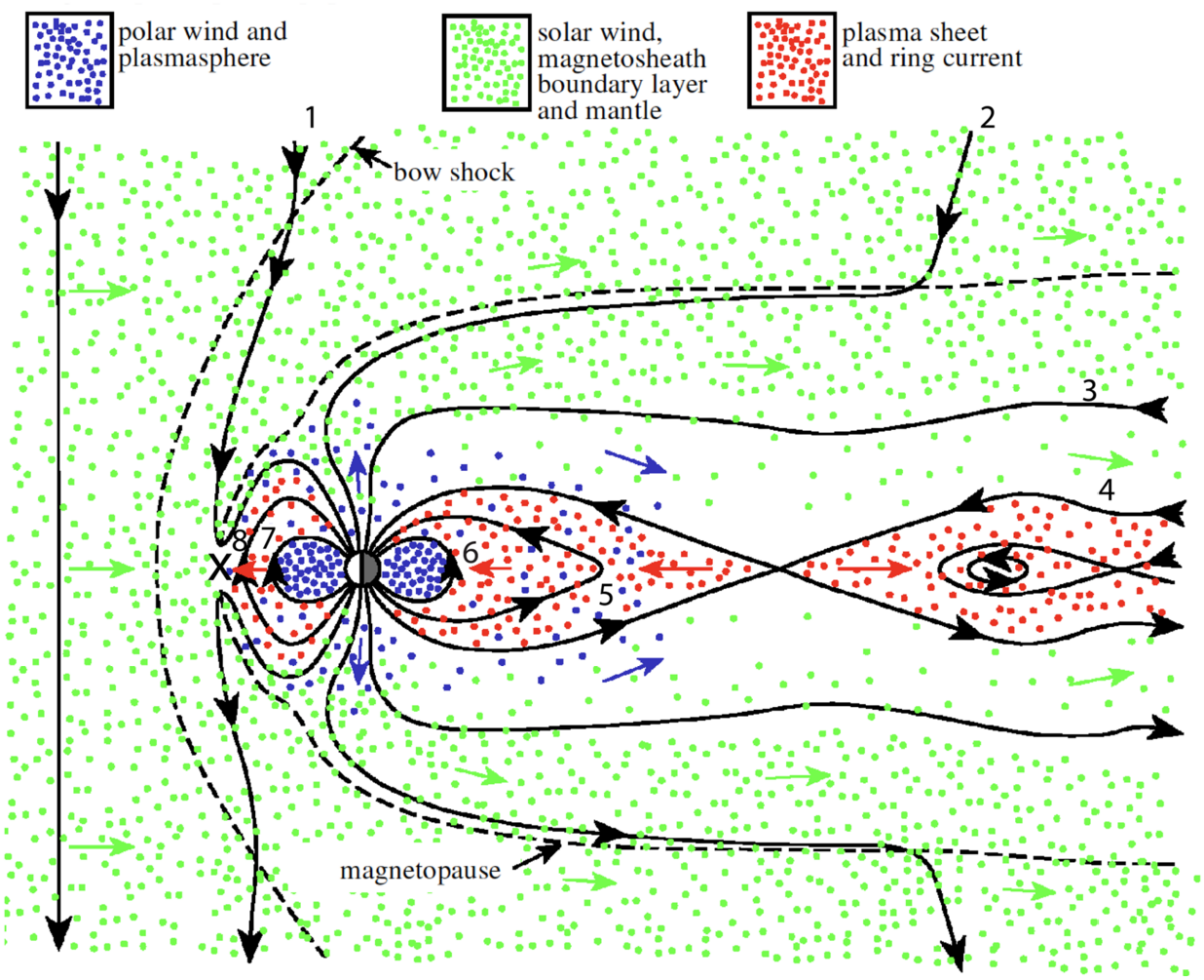
The Dungey cycle showing the motion of the field lines after reconnection (marked with a black cross) on the dayside of the magnetosphere (line 1), motion through the lobes (lines 2 and 3), reconnection in the tail (line 4) and motion towards Earth (line 5 and 6), and back to the dayside of the magnetosphere (line 7 and 8). The dots show the plasma populations from the solar wind (green) and the ionosphere (blue) which form the population of the tail after energization (red). The short colored arrows show the motion of the plasma. Adapted from Cowley et al. (2002) and Dungey (1961)

The coupling between the Sun and the Earth is done mainly through reconnection between the magnetic field of the solar wind and the Earth magnetic field (Dungey 1961). Other processes have been proposed in the past such as viscous interaction (Axford and Hines 1961) but these are now generally considered to be secondary effects. The solar wind magnetic field is varying on time scales of minutes, hours or days depending on its origin on the sun and the solar activity. On the other hand, the Earth’s magnetic field is fairly constant, especially around the magnetopause, until reconnection with the solar wind magnetic field occurs and start to modify it and forces the motion of the field lines. Such motion for solar wind magnetic field pointing Southward is shown on Fig. 2. The location where the solar wind magnetic field is opposite to the Earth magnetic field is the subsolar point where reconnection starts and cuts the solar wind magnetic field line in two parts (line 1), the northern part of the field line is now connected to the North hemisphere of the Earth and the Southern part to the South hemisphere. Solar wind particles (green) can now go through the magnetopause (dashed line) and enter the magnetosphere. As the solar wind plasma move downstream the solar wind plasma populates the lobes of the magnetosphere (lines 2 and 3). Then both lobes field lines are pushed towards each other and reconnect in the magnetotail (line 4). After reconnection the field lines convect towards Earth (lines 6 and 7) and move to the dayside to close the cycle (lines 7 and 8). More recently Dai et al. (2024) showed that under certain conditions the sunward convection (parts 7-8) may be driven with dayside reconnection only, allowing a much fast response of the geomagnetic activity to the interplanetary magnetic field (IMF) turning Southward.

The parameters which control the rate and location of reconnection are still not fully understood. The shear between the IMF and the terrestrial magnetic fields (e.g. Trattner et al. 2021), solar dynamic pressure change (Boudouridis et al. 2007), or plasma $\beta $ have all been suggested as possible controlling mechanisms. Phan et al. (2013) proposed that a large jump in $\beta $ across the magnetopause may prevent reconnection. Walsh et al. (2014) provided evidence for such an effect with observations that showed the rate of magnetopause reconnection was suppressed when the cold, high density plasmaspheric plume reached the magnetopause. Such effects are usually observed only at one point in space and cannot be studied over a large portion of the magnetopause simultaneously. SMILE will observe the magnetopause position over a large distance in the Y_GSE_ direction (up to 8 R_E_), using the soft X-ray instrument (SXI) and give information on the position and motion of the magnetopause at large scale to identify regions where reconnection may be taking place.

**Fig. 3:**
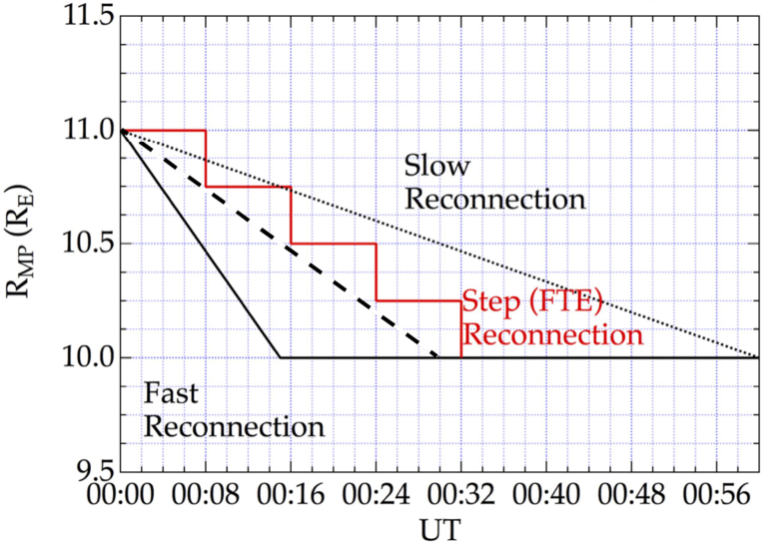
Subsolar magnetopause motion due to reconnection. Fast reconnection (solid line), normal reconnection (dashed line) and slow reconnection are sheon. In addition pulse reconnection with a pulse of reconnection every 8 minutes is shown in red. (adapted from Sibeck et al. 2023)

One of the key unanswered questions in magnetospheric physics is whether dayside reconnection is variable, occurring in pulses (Lockwood and Smith 1992) separated by approximately 10 minutes or stable and constant (Phan et al. 2004) that can last for hours. Most likely, both occur but under different solar wind conditions. A simple method to measure changes in the reconnection rate is to measure the motion of the magnetopause, or in other words its position as a function of time. Figure 3 shows a few types of reconnection, in which the magnetosphere erodes inward by about 1 R_E_ in 15 minutes (fast reconnection), or in 1 hour (slow reconnection) or in about 30 minutes (normal reconnection). All the three rates assume continuous and steady reconnection. Reconnection could however be intermittent or pulsed and the magnetopause will move in steps. Figure 3 shows a reconnection with pulses separated by 8 minutes with each step eroding the magnetopause by 0.25 R_E_ (red line).

The SMILE SXI instrument will image the magnetopause continuously on minute time scales.

To distinguish these models from each other we placed requirements on the SXI instrument as follows:

- For solar wind flux > 4.9 × 10^8^/cm^2^s, the location of the subsolar magnetopause shall be determined with a better accuracy than 0.5 R_E_ and better than 5 min time resolution from 20 R_E_ geocentric (requirement);
- For solar wind flux > 1.6 × 10^9^/cm^2^s, the location of the subsolar magnetopause shall be determined with an accuracy of 0.125 R_E_ and at 1 min. time resolution 20 R_E_ geocentric (goal).

The requirements set out the minimum acceptable performance that the instruments must meet to address the SMILE science questions, however the mission team also set goals for instrument performance to push the instrument capabilities and increase the science return. Distinction between the models as shown on Fig. 3 would be possible if the measurement goals are met. The requirement would be achieved with the expected SXI performances (see Sembay et al. 2025, this collection) and we may be able to distinguish the different models under certain conditions, for instance for a larger erosion (a few R_E_) or for stronger solar wind flux. SMILE will be able to image the position of the magnetopause as a function of time and should therefore be able to distinguish between the various modes of reconnection.

**Fig. 4:**
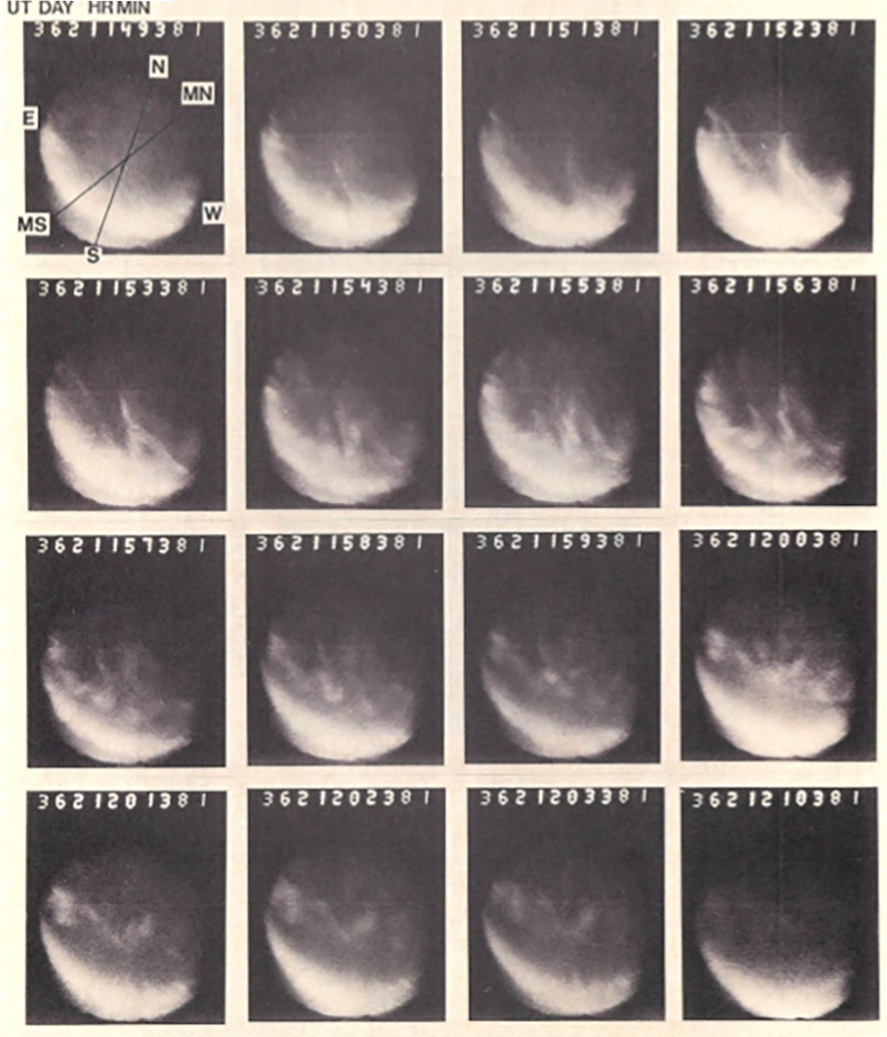
Auroral images taken from the ground at 630.0 nm on December 27, 1984 between 11:49 and 12:10 UT. Geographic (N-S) and geomagnetic (MN-MS) north-south directions are indicated. Although the main auroral arc is moving southward, bright patches are moving poleward (poleward moving auroral forms). Image reproduced with permission from Sandholt et al. (1986), copyright by AGU

The reconnection rate can also be estimated from the auroral emissions on the dayside of the Earth, with the auroral cap indicating the open magnetic flux area. Poleward moving auroral forms (PMAFs) are observed on the dayside aurora as bright patches that move poleward (Sandholt et al. 1986) with a lifetime of 5-10 min and a recurring frequency of 6 min (Fasel 1995). Figure 4 shows the poleward moving auroral form that brightened and move poleward before fading after 5-10 min. They are believed to be the ionospheric signature of magnetic reconnection at the magnetopause. The UV instrument (UVI) on SMILE has been specifically designed to minimize the signal from dayglow so as to image the dayside aurora.

The requirements put on the UVI instruments on the dayside aurora and poleward moving structures are:

- Transient brightenings of 100 R or more in the dayside aurora shall be measured with a time resolution of at least 1 min (requirement);
- Transient brightenings of 20 R or more in the dayside aurora shall be measured with a time resolution of at least 15 s (goal);
- The position of features in the aurora shall be measured with an accuracy of at least 150 km at 1 min time resolution below 18 R_E_ altitude and at least 170 km at 1 min resolution above 18 R_E_ altitude (requirement);
- The position of features in the aurora shall be measured with an accuracy of at least 50 km at 15 s time resolution from 19 R_E_ altitude (goal).

The polar cusp is a key region of coupling between the solar wind and the magnetosphere. It is the first point of direct entry of solar wind particles into the magnetosphere and ionosphere and its size, location, and indeed the number of cusps (Wing et al. 2001) are highly dependent on the plasma and magnetic field conditions in the solar wind. It has been demonstrated with many spacecraft and from the ground that the polar cusp moves in latitude as the IMF Bz component changes. It is found at lower latitude when IMF is southward and at higher latitude when the IMF is Northward (e.g. Newell et al. 1989). This effect is explained by the process of reconnection on the dayside equatorial magnetopause for southward IMF and on the high latitude lobes when IMF is northward (e.g. Escoubet et al. 2008). Observing the cusp position and its motion will therefore address the question of the fundamental modes of the dayside solar wind/magnetosphere interaction and if reconnection is pulsed or continuous.

**Fig. 5:**
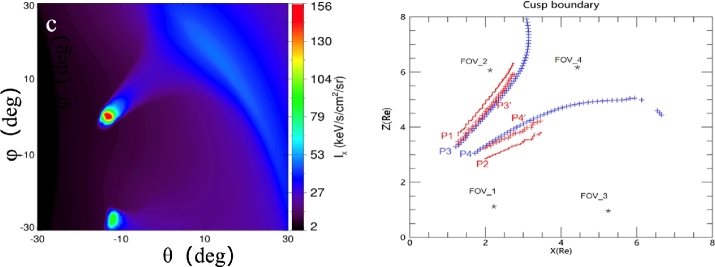
Left: the polar cusps seen in soft X-ray using an MHD model (Hu et al. 2007). The mid-altitude cusp is the brightest region due to high solar wind density plasma and high geocorona density, as compared to the exterior cusp or the magnetosheath. Right: The equatorward and poleward boundaries of the Northern cusp from the MHD model (blue crosses), from the soft X-ray image (red dots) and from the soft X-ray + cusp eliptic cross-section (red crosses) (Connor et al. 2025, this collection, or Wang et al., private communication)

MHD simulations have shown that the cusp, in particular the mid-altitude cusp, can be bright in soft X-ray (Sun et al. 2019). Figure 5 left shows a soft X-ray simulated image of the polar cusp (red spot at mid-altitude) and magnetosheath (dark blue region). Using this image, the boundaries of the cusp can be extracted (see method in Sun et al. 2021) and these are shown as the two lines with red dots (poleward boundary P1 and equatorward boundary P2) in the right panel. The cusp boundaries obtained from the MHD model use the plasma pressure to identify the poleward (P3) and equatorward boundaries (P4). As can be seen there is a fraction of an R_E_ difference between P1-P2 and P3-P4. This come from the fact that the cusp cross-section is not a circular shape but more an ellipse, elongated in local time (see Newell and Meng 1992). Assuming that the cusp is an ellipse, the boundaries obtained from the X-ray image P3’ and P4’ are then much closer to the MHD simulations boundaries (Fig. 5 right).

To measure the cusp position and motion the following requirements were put on the SXI instrument:

- The poleward and/or equatorward edges of the mid-altitude cusp shall be determined with a spatial resolution of at least 0.25 R_E_ and a time resolution of at least 5 min for solar wind flux > 8 × 10^8^/cm^2^s from 8 R_E_ distance from SXI to the Northern cusp (requirement).
- The poleward and/or equatorward edges of the mid-altitude cusp shall be determined with a spatial resolution of at least 0.1 R_E_ and a time resolution of at least 1 min for solar wind flux > 1.6 × 10^8^/cm^2^s (goal).

To complement these observations of the magnetopause, cusp and dayside aurora and make the SMILE mission self-standing, an ion instrument and a magnetometer are included in the payload. Measurements accuracy has also been required on these instruments as follows:

- H+ moments (density, velocity and temperature) shall be measured at 2 min. or better time resolution with at least 20% accuracy (requirement);
- H+ moments (density, velocity and temperature) shall be measured at 2s or better time resolution with at least 20% accuracy (goal);
- H+ velocity shall be measured up to 800 km/s (requirement);
- H+ velocity shall be measured up to 1000 km/s (goal);
- The three components of the magnetic field B shall be sampled with at least 2 min. time resolution, 0.1 nT resolution in the solar wind and an absolute accuracy of 2 nT (requirement);
- The three components of the magnetic field B shall be sampled at 8 samples/s or better and 0.1 nT resolution in the solar wind and an absolute accuracy of 0.5 nT (goal).

Since the observation of the magnetopause and cusp with the SXI instrument requires a minimum strength of the solar wind flux we estimated the number of events when the solar wind flux is above this threshold. We used measured solar wind data from the previous solar cycle for such estimation, assuming persistence. We assumed that the SMILE mission will start its operations in 2026 and will last 3 years (2026-2028). With a solar cycle period of 11 years, this would correspond to the years 2015-2017 in the previous solar cycle.

**Fig. 6:**
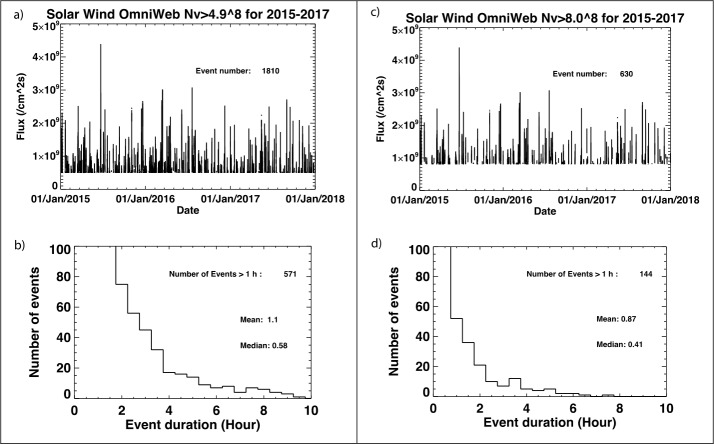
Solar wind flux ($nV$) from OMNI solar wind data (King and Papitashvili 2005) during the 2015-2017 timeframe. All events with nV > 4.9 × 10^8^ cm^−2^ s^−1^ and nV > 8 × 10^8^ cm^−2^ s^−1^ are shown on panel a and c respectively. The total number of events is indicated in the plot. Panel b and d show the histogram for the events. The number of events longer than 1 h are indicated as well as the mean and median values for all events

Figure 6 shows the number of events (one event is counted when a solar wind/geomagnetic parameter is above a threshold for at least 5 minutes) that were observed in 2015-2017 with a solar wind flux above 4.9 × 10^8^ cm^−2^s^−1^ (moderate flux) and 8 × 10^8^ cm^−2^s^−1^ (strong flux). The total number of events were 1810 for moderate flux and 630 for high flux. If we select only events where the flux was higher than the above thresholds for more than one hour, we obtain 571 and 144 events which are adequate for statistical analysis. These events added together take respectively about 10% and 2.5% of the time over the 3 year mission. The moderate flux is the minimum threshold to measure the magnetopause position from SXI images at 5 minutes time resolution and the strong flux is required to measure the position of the cusp boundaries (see above science requirements). Using the subsolar magnetopause and Northern and Southern hemisphere cusp observing time (A. Read private communication or Sembay et al. 2025, this collection) to be around 41%, 45% and 22% respectively over the three years period, we would obtain 96 events with the Northern or Southern cusp (at least one boundary) and 234 events with the subsolar magnetopause.

### What Defines the Substorm Cycle?

Although aurora drawings may have been found in prehistoric caves from as early as 30,000 BC, the earliest written reports date from 977-957 BC in the history of ancient China. Auroras are found around the magnetic poles of the Earth magnetic field. Nowadays their location is in the Northern and Southern hemisphere polar regions but the locations could have changed over a few 10,000s years. The last excursion of the geomagnetic pole across the equator and back to its original position took place around 42,000 years ago. Such an excursion would have certainly moved the aurora to very low latitudes.

**Fig. 7:**
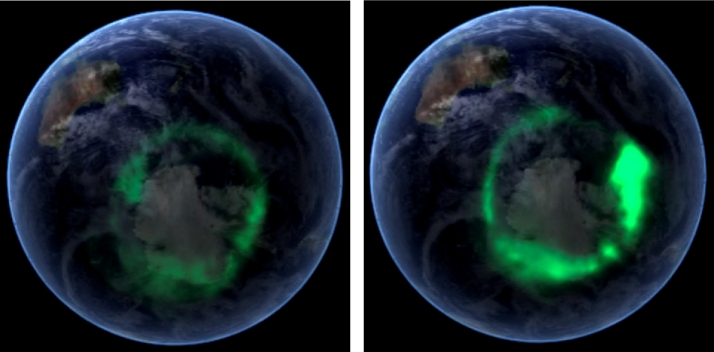
Auroral oval image (green) taken by the IMAGE spacecraft on 11 September 2005. This image was taken over the Southern pole (aurora australis) where we can observe antartica underneath and Australia in the upper left corner

Auroras are made of small (a few kilometers) and large (a few hundreds of kilometers) arcs that, together, form an oval approximately centered around the geomagnetic pole. Figure 7 shows the auroral oval (green) centered around the Southern geomagnetic pole. The auroral oval during quiet time is shown on the left panel. Although we can clearly see the oval, the intensity of the auroral emission is low. On the other hand, on the right panel, the auroral oval a few minutes later is brighter and more active. The largest and most active part of the auroral oval (right and bottom side) is located on the nightside of the Earth and its sudden brightening and poleward expansion is what is called an auroral substorm (Akasofu 1964).

**Fig. 8:**
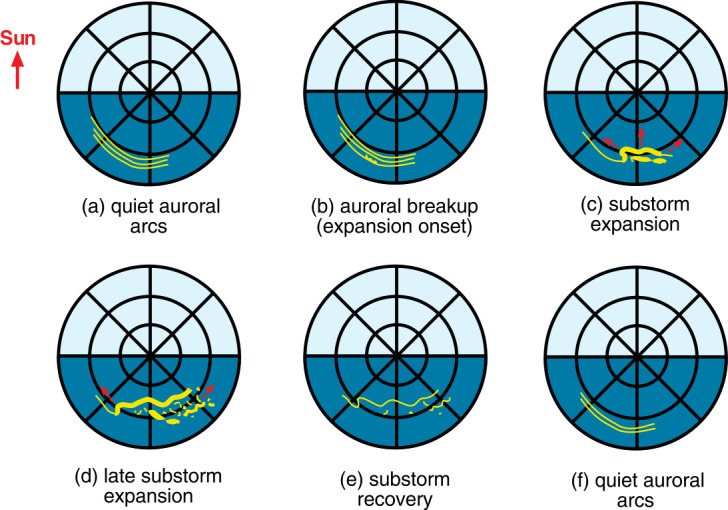
Sketch of auroral intensification during a substorm (A. Lui, private communication, after Akasofu 1964). Yellow lines shows the main auroral emissions on the nightside. Geomagnetic latitudes are marked by circles separated by 10 degrees. Red arrows show the motion of arcs during the substorm expansion

Akasofu (1964) first sketched the substorm development by recording images of the Northern light. Although many papers and special conferences studied the physical process responsible for a substorm, the decription of the auroral oval changes triggered by a substorm has not changed significantly (Fig. 8). Before substorm quiet arcs are located on the nightside approximately parallel to each other (Fig. 8a) to form the auroral oval. Then one of the auroral arcs, typically the most equatorward, starts to break-up (Fig. 8b). Then the auroral arcs brighten and expand poleward as well as westward and eastward (Figs. 8c and 8d). About 30 minutes later, the expansion stops, and the aurora light decreases and start to retreats equatorward during substorm recovery phase (Fig. 8e). Finally, at the end of the substorm, the aurora is again formed by a few parallel quiet arcs.

The magnetospheric counterpart to the auroral substorm is characterized by a large-scale deformation in the magnetosphere, most notably the sudden cross-magnetotail current and its diversion into and through the ionosphere in the form of a substorm current wedge (McPherron et al. 1973). The canonical magnetosphere substorm is broken down into three phases: the growth phase, during which dayside reconnection adds magnetic energy into the magnetotail lobes (McPherron 1970); the expansion phase, during which the magnetotail rapidly dipolarizes and magnetic flux is closed; the recovery phase, during which the dipolarization subsides, open flux is ejected downtail in a plasmoid (e.g. Hones 1979) and distinct aurora forms, such as omega bands, propagate through the dawnside auroral region (e.g. Forsyth et al. 2020). During a substorm, the magnetotail stores energy and then releases it towards Earth to produce the brightening and fast motion of the nightside auroras (Akasofu 1964). These bright aurora are associated with an enhanced westward electrojet in the ionosphere, the signature of which is sometimes used to identify substorms (Newell and Gjerloev 2011; Forsyth et al. 2015). Their duration is typically a few hours and their recurrence can vary from a few hours to a few days (Borovsky and Yakymenko 2017).

The science requirement on both UVI and SXI instruments to address the substorm cycle are the following:

- For solar wind flux >3.6e8 /cm^2^s, the location of the subsolar magnetopause shall be determined with a spatial accuracy better than 0.5 R_E_ and a time resolution better than 10 min. from 20 R_E_ geocentric
- The poleward and equatorward boundaries of the auroral oval shall be identified at all local times with a spatial resolution of at least 300 km at 1 min. time resolution from 19 R_E_ altitude.
- Transient brightenings of 100 R or more in the aurora shall be measured with a cadence of at least 1 min.

**Fig. 9:**
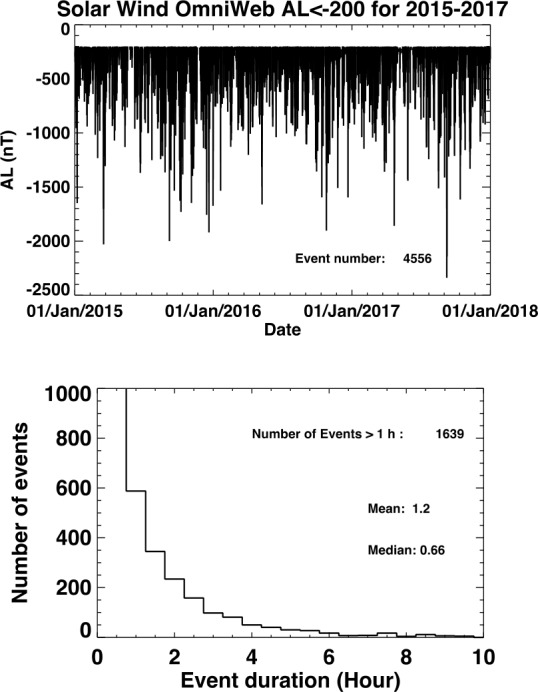
Number of events when AL index is below −200 nT during 2015-2017 from OMNI data (King and Papitashvili 2005). The total number of events is 4556 and the number when AL is below −200 nT during more than 1 h is 1639. The average number of events above 1 h would be about 1.5 per day

Substorms typically occur a few times a day. We investigated the number of substorms that would be expected during the 3 years SMILE mission. We use the AL index from OMNI database between 2015 and 2017 and use AL below −200 nT to define a substorm. Figure 9 shows that 4556 substorms occurred out of which 1639 were longer than 1 h. Such a high number would be sufficient to make statistical analysis of substorm characteristics and properties.

### How do CME-Driven Storms Arise and What Is Their Relationship to Substorms?

Geomagnetic storms are large disturbances in the Earth magnetic field typically caused by Coronal Mass Ejections (CME) or corotating Interaction Regions (CIR) produced at the Sun and propagating in the interplanetary space (Borovsky and Denton 2006). CME-driven storms occurred mainly during solar maximum while CIR-driven storms occur in the descending phase of the solar cycle. Geomagnetic storms occur around once a month during solar max and the descending phase (Gonzalez et al. 2007).

Geomagnetic storms are defined by a large change in the Earth magnetic field, measured on the ground by four stations spread around the equatorial plane of the Earth. This effect is due to the strong enhancement of the ring current flowing around the Earth in the equatorial plane at a distance of a few Earth radii. Storms are characterized by a sudden increase of the horizontal (H) component (with an increase up to 100 nT in a few minutes) of the equatorial magnetic field, known as the storm sudden commencement, followed by a large decrease of the H component (main phase) during 2-8 hours and finally a slow increase of the H component to its original values (recovery phase) that can last from 8 hours up to 7 days (Gonzalez et al. 1994). After Gonzalez et al. (1994), geomagnetic storms are typically defined to have occurred if Dst drops below −50 nT. The larger the decrease in the H component (it can reach −300 nT or less for extreme storms), the stronger the geomagnetic storm is. Tsurutani et al. (1992), studying a few great storms showed that they were associated with a solar flare on the sun and a high speed stream, associated with a shock, traveling towards Earth in the interplanetary medium. They showed that it is the extreme interplanetary magnetic field southward component (Bz between −10 and −50 nT) that was driving the strength of the geomagnetic storms.

**Fig. 10:**
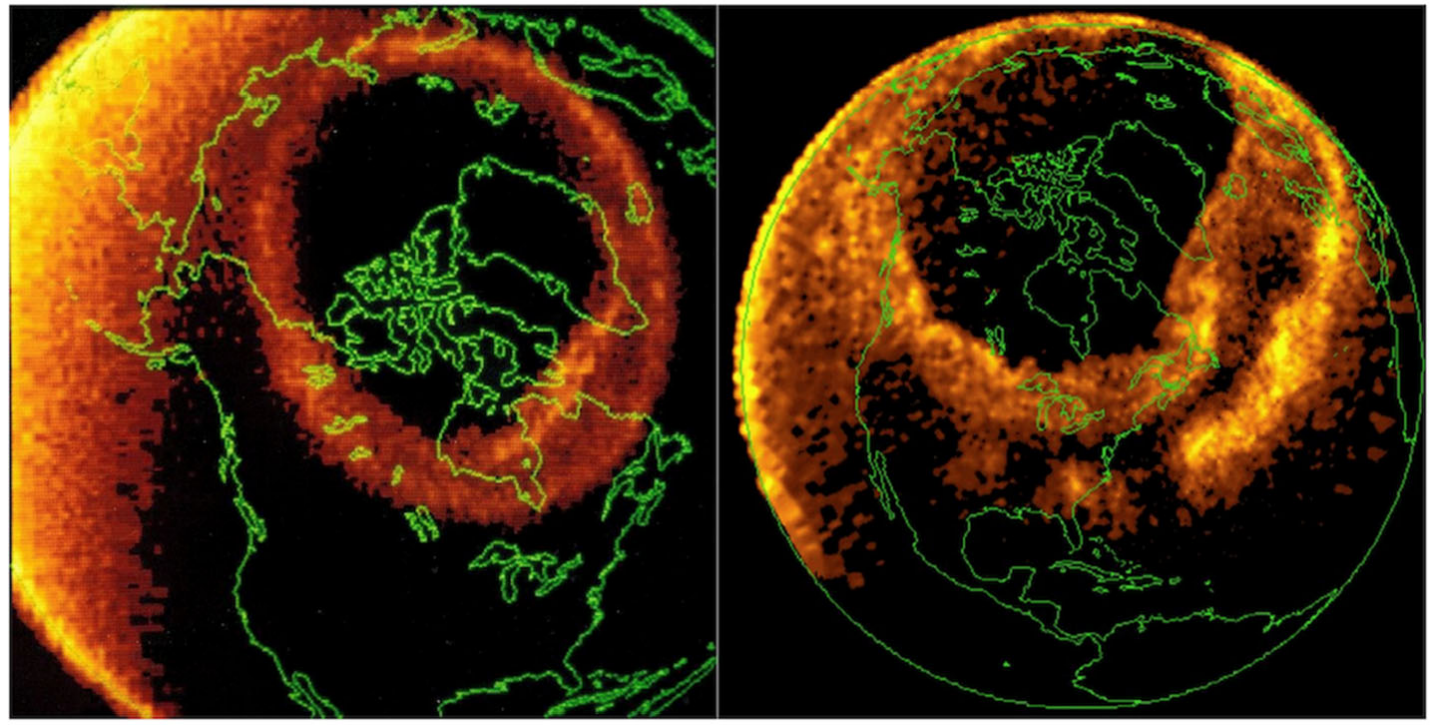
NASA Dynamic explorer 1 image of the auroral oval under normal conditions (left) and during the great geomagnetic storm of 13-14 March 1989 (right). The right image was taken in the Southern hemisphere and mapped on the Northern hemisphere (from Johnsen 2013). Note that the scale is enlarged on the left side due to the spacecraft being closer to Earth and the auroral oval is smaller under normal conditions than during geomagnetic storm. The Sun direction is in the upper-left corner

Together with large change in Earth’s magnetic field, geomagnetic storms produce very intense aurora with the auroral oval expanding to more equatorward latitudes than normal, reaching sometimes the Southern part of Europe or the United States in the case of the northern hemisphere oval. Figure 10 shows the auroral oval during normal conditions and during the great geomagnetic storm of March 1989 (Dst $=-597$ nT). This geomagnetic storm caused a nine-hour outage of Hydro-Québec’s electricity transmission system. The collapsed power grid left six million people and the rest of Quebec without electricity for hours on a very cold night (Boteler 2019 and reference therein). United States, United Kingdon and Sweden also had problems with their power grid. This geomagnetic storm was produced by two CMEs following each other within 2 days. This succession of CME produced an extreme storm since the first CME “cleaned” the solar wind allowing the second one could to travel at full speed without much deceleration and impact the Earth with best efficiency (e.g. Boteler 2019).

The auroral oval was also greatly affected as shown on Fig. 10 right picture.

Under normal conditions, the auroras are located over the Northern part of Canada (Fig. 10 left picture) and under extreme storms it can reach Florida in the US (Fig. 10 right picture). On the upper right corner of the pictures, in Europe, the aurora is in Northern Scandinavian countries under normal conditions and can reach Spain when the geomagnetic storm occurs.

SMILE will shed new light on geomagnetic storms by measuring over up to 40 hours continuously the magnetopause position and its extreme motion towards Earth (sometimes inward of the geostationary orbit). It will also measure the expansion and brightening of the auroral oval to measure the polar cap area and the energy that is transferred to Earth in the solar wind-magnetosphere coupling.

The science requirements on both the UVI and SXI instruments to address the CME-driven storms are the following:

- For solar wind flux > 3.6 × 10^8^ /cm^2^s, the location of the subsolar magnetopause shall be determined with a spatial accuracy better than 0.5 R_E_ and a time resolution better than 10 min. from 20 R_E_ geocentric
- The poleward and equatorward boundaries of the auroral oval shall be identified at all local times with a spatial resolution of at least 300 km at 1 min. time resolution from 19 R_E_ altitude.
- 2000 R changes in brightness of 30 kR aurora shall be identified and measured with a cadence of at least 1 min.

## Instrumentation

To address the science objectives, four instruments have been integrated on the SMILE spacecraft. The 3D ion instrument and the magnetometer will measure the main characteristics of the plasma just outside the magnetosphere before it impacts the magnetosphere. Simultaneously the soft X-ray and UV imagers will measure the changes that the solar wind produces in the magnetosphere. Table 1 summarises the main characteristics of the four instruments.

**Table 1:** Instruments on SMILE

Instrument	Measurement	Mass	Power	Principal Investigator
Light Ion Analyser (LIA)	3D ion distribution function of 5 eV-25 keV at 250 ms.	2 × 3.0 kg	2 × 8 W	DAI Lei, NSSC, CAS, China
Magnetometer (MAG)	3 components of magnetic field up to 40 Hz	8.8 kg (incl. 3m boom of 6 kg)	7 W	LI Lei, NSSC, CAS, China
Soft X-ray Imager (SXI)	X-ray 0.2-2.5 keV with 15.5° × 26.5° FOV and a few minutes resolution	33 kg	38 W	Steve Sembey, Leicester U., UK
Ultraviolet imager (UVI)	UV 160-180 nm with 10° FOV and 1 minute resolution	15.5 kg	40 W	ZHANG Xiaoxin, NOSW, CMA, China

**Fig. 11:**
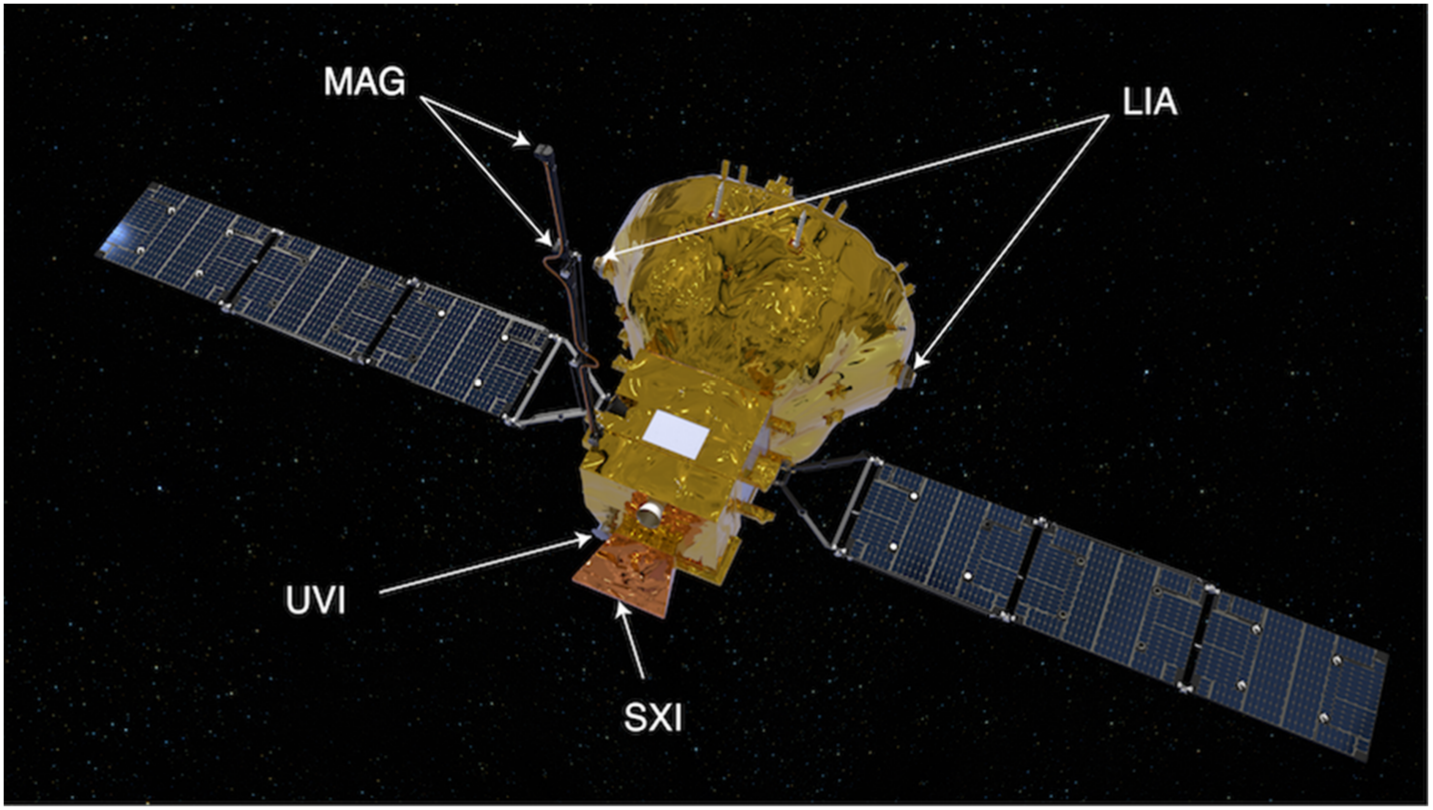
SMILE spacecraft and instruments in flight configuration. The ion instrument two sensors (LIA), magnetometer two sensors (MAG), soft X-ray imager (SXI) and UV imager (UVI) are shown (Work performed by ATG under contract to ESA)

The spacecraft is a 3-axis stabilized spacecraft to allow the imagers to point toward their targets (Li et al. 2024, this collection). The Light Ion Analyser (LIA) consists of two identical semi-hemispheric sensors, located on opposite sides of the spacecraft (Fig. 11) that allow the measurement of the full 3D distribution function (4$\pi $) at up to 250 ms cadence (Dai et al. 2025, this collection). The energy range is from 5 eV to 25 keV split into 62 steps. The 3D distribution functions will be downloaded to the ground where the moments (density, velocity, temperature, and other parameters) will be computed.

The magnetometer (MAG) is made of two tri-axial fluxgate magnetometers located on a 3 m deployable solid boom (Fig. 11) (Li et al. 2025, this collection). One sensor will be located at the tip of the boom and the other 80 cm in-board. They will measure the magnetic field up to a frequency of 40 Hz. The two magnetometers are used to remove the perturbations created by the spacecraft and for redundancy in case of failure of one of them. To calibrate the sensors and remove offsets, the spacecraft will be slowly rolled at regular intervals of a few months in the lobes of the magnetosphere.

The soft X-ray instrument (SXI) is a wide field-of-view (FOV) lobster-eye instrument that will measure soft X-ray in the range 0.2-2.5 keV (Fig. 11) (Sembay et al. 2025, this collection). The FOV is 15.5° × 26° with the long edge approximately perpendicular to the Sun-Earth line. The energy resolution is 50 eV. Two large CCDs (8.12 × 8.12 cm) will measure the soft-X ray photons, detecting the position, energy and time of arrival of every X-ray photon that strikes the CCD during operations. These photon event lists will then be integrated on the ground to create images of the magnetosheath and polar cusp, its main science targets. The large FOV will enable the magnetopause to be imaged over a distance of up to 8 R_E_, perpendicular to the Sun-Earth line. With SMILE very eccentric orbit (see below), SXI will image the dayside magnetosphere for more than 40 hours continuously. SXI will be operating above 50,000 km, outside of the radiation belts, when the spacecraft is most of the time in the magnetospheric lobes and the magnetosheath.

The Ultraviolet imager (UVI) will image the auroral oval between 160 nm and 180 nm. It is using four mirrors and its FOV is 10° (Zhang et al. 2025, this collection). It is located next to the SXI instrument with its pointing direction making an angle of 24 deg. with respect to SXI, pointing in the Earth’s direction (Fig. 11). UVI will always point to the North pole such as to image the Northern auroral oval for more than 45 h continuously. Its minimum operation altitude, not to be affected by radiation belts particles, is 30,000 km. Special filters have been developed to suppress as much as possible the dayglow and enable detection of the dayside aurora and derivation of the boundaries of the auroral oval under all daylight illuminated conditions.

## Orbit and Pointing Strategy

**Fig. 12:**
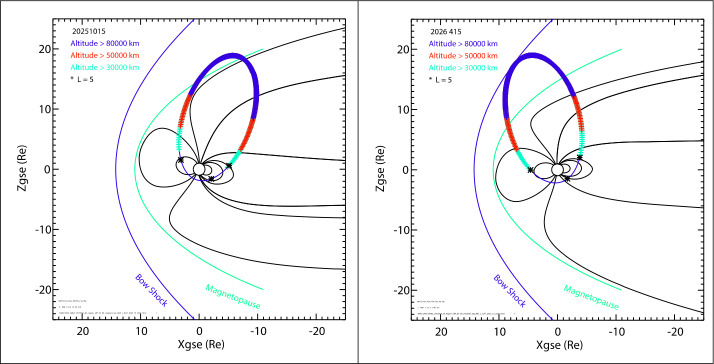
SMILE orbit in fall (left panel) and spring (right panel). The orbit is shown with a blue thin line and altitude above 80,000 km in blue, altitude above 50,000 km in red and altitude above 30,000 km in green. The points where the McIlwain parameter L = 5 is shown with an asterisk. Solid black lines line represent field lines from the Tsyganenko 1996 model (Tsyganenko and Stern 1996). Finally, the bow shock (Merka et al. 2005) and magnetopause (Sibeck et al. 1991) are shown in blue and green lines. The solar wind parameters used for these models are N = 5 cm^−3^, $V=400$ km/s, B = 5 nT

SMILE will be launched by an Arianespace Vega-C launcher from Kourou, French Guiana. The launcher will inject the spacecraft in a low altitude orbit around 450 × 700 km. The spacecraft will then use its on-board fuel (70% of a total mass of 2.3 tons) to reach its nominal orbit of 1.8 × 20 R_E_ geocentric distance with 70° inclination. Figure 12 shows the final orbit on 15 October 2025 and on 15 April 2026. During October, SMILE will stay in the magnetosphere with the apogee just reaching the magnetopause, while in April, SMILE will stay most of the time in the magnetosheath around apogee. The solar wind would only be accessed for very short time during normal solar wind conditions, with this time increasing during period of high solar wind dynamic pressure. The colors along the orbit show when SXI will be operating above 50,000 km (red and blue) and when UVI will be operating above 30,000 km (green, red and blue).

LIA will be operating when L > 5 (marked with an asterisk) and it will be put in standby or off during the crossing of the radiation belts. MAG instrument will be operating all along the orbit.

Around perigee, at 5000 km of altitude, the spacecraft attitude will be changed to point the communication antenna towards the O’Higgins ground station in Antarctica. The Sanya ground station in China will also be used during nominal operations. Using high data rate X-band, the spacecraft should be able to dump 34 Gbit of data every orbit during a communication pass of 10-15 minutes.

The pointing strategy was developed early during the mission design to take into account all constrains of the spacecraft and instruments as well as the science targets. The baseline pointing is driven by SXI science targets and constraints: the SXI line of sight will point towards the +X_GSE_ axis with an angle of 20.3 deg. away from the Earth disk edge. This is approximately pointing SXI towards the magnetopause subsolar point at 10 R_E_ distance from Earth. This pointing will be used above 50,000 km. Between 30,000 km and 50,000 km, the spacecraft will point according to UVI requirements and constraints: the UVI line of sight will point at +80° geographic latitude to maximize the observation of the auroras in the North hemisphere. Below 3000 km the pointing will maximize the solar array output power, except during communications where the X-band antenna will be pointing towards the ground station.

## The SMILE Working Groups

**Fig. 13:**
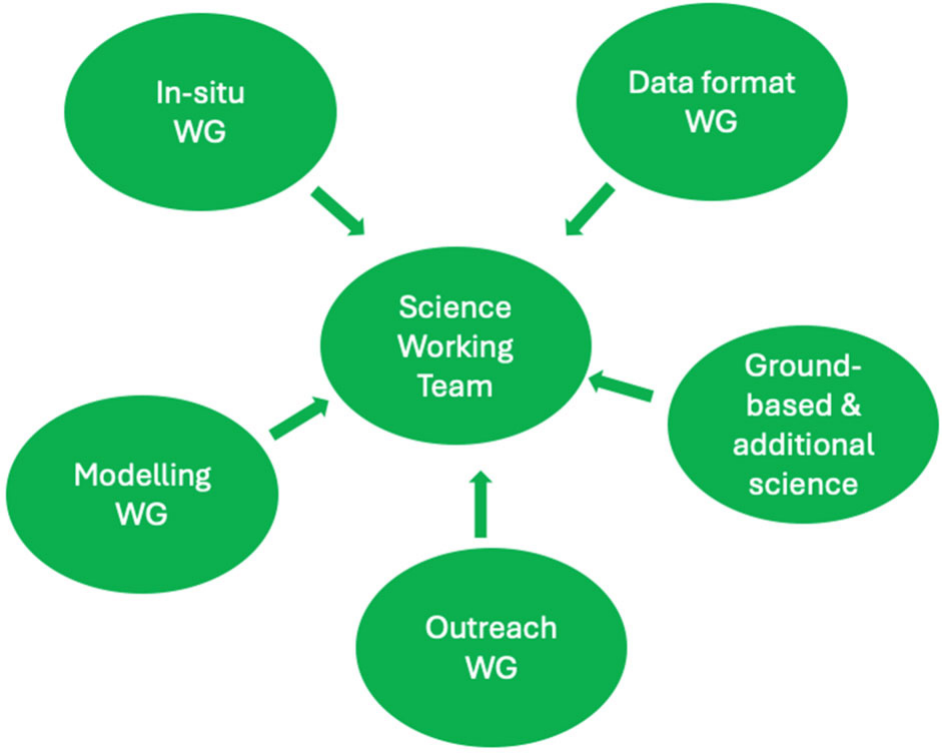
The five SMILE working groups formed by the science working team to prepare SMILE science future investigations

The Science Working Team appointed five working groups to prepare the mission with modeling and data analysis (Fig. 13):

*Data Formats Working Group (DFWG)* (Chair: Catarina Alves de Oliveira, ESAC, Spain (up to 2023); Rocio Guerra (from 2024), ESAC, Spain)

This working group has been formed to define the data products and supporting data sets, as well as their format. There will be two archives for distribution of data to the user community, one in ESA and one in CAS. The exchange of data between the two archives as well as the quicklook plots design is also addressed by the group.

*Ground-Based and Additional Science Working Group (GBASWG)* (Chair: Jenny Carter, Leicester University, UK)

This working group makes the link with ground-based observatories and coordinates the operations when SMILE will be operating. Preliminary contacts with operational and forthcoming space missions are taking place in order to coordinate observing and software efforts. SMILE tools are being developed to coordinate ground-based observations together with SMILE in-situ and imaging observations.

*In Situ Working Group (ISWG)* (Chair: Lei Dai, NSSC/CAS)

The SWG activity is centered on optimising the design, operations and calibrations of the in situ instruments. Coordination with other in-situ missions such as Arase, MMS and THEMIS are done based on science regions observed and conjunctions between the spacecraft.

*Outreach Working Group (OWG)* (Chair: Graziella Branduardi-Raymont (up to 2023) and Colin Forsyth (from 2024), MSSL/UCL)

This working group promotes the SMILE mission to various public audiences from primary and secondary schools as well as general public and user community. Presentations and workshops are organized to share the SMILE science. This group also collects images or videos taken during various spacecraft and instrument activities.

*Modelling Working Group (MWG)* (Co-chaired: Hyunju Connor (Univ. Alaska Fairbanks, Andrey Samsonov (MSSL, UK) and Tianran Sun, NSSC/CAS, Beijing, China)

This WG aims to coordinate and drive the simulation and modelling activities taking place in SMILE collaborating institutes and leading to predict the soft X-ray images which SXI will generate. This group is simulating the changes which can be expected in magnetospheric boundary locations under differing solar wind conditions and also investigates how to extract magnetospheric boundaries and cusp positions from SXI simulated images. The WG also addresses UV imaging modeling in particular the sources of particles producing the aurora.

The working groups meet at regular intervals, most of the times online, and in person a few times a year. They report their activities during the SWTs, held twice a year. The data format working group activities are reported by Guerra et al. (2025, this collection), the Ground-Based and Additional Science Working Group activities are reported by Carter et al. (2025, this collection) and the Modelling Working Group by Connor et al. (2025, this collection).

## SMILE and Other Missions

SMILE will be very complementary to other magnetospheric missions such as Arase, MMS, THEMIS and Swarm since it will image the regions where other missions will be taking measurements in-situ. It is also very well aligned with the upcoming Carruthers mission, which will image Earth’s geocorona/exosphere from the Sun-Earth L1 point and is due for launch in mid-2025. The TRACERS mission, to be launched in 2025, will study the motion of the polar cusp at low altitude using two identical spacecraft and will complement SMILE images of the mid-altitude and exterior cusp.

In addition SMILE will also cross the magnetopause, almost every orbit, depending on the season at high latitude due to its high inclination and eccentric orbit. We have investigated when THEMIS A, D, E and SMILE would be crossing the magnetopause within a distance of +/−1 R_E_ using SSCweb (https://sscweb.gsfc.nasa.gov) orbit tools.

**Fig. 14:**
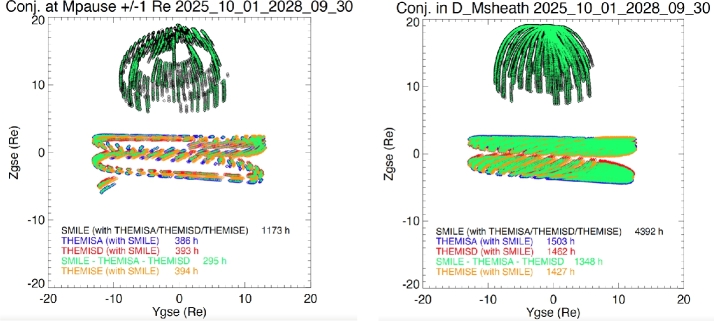
Position of magnetopause (left) and magnetosheath (right) crossings with SMILE (black diamonds), THEMIS A (blue diamonds), THEMIS D (red diamonds) and THEMIS E (orange diamonds in YZ_*GSE*_ plane.). The crossings when both SMILE, THEMIS D and THEMIS E are simulatenously at the magnetopause are shown in green diamonds. The number of hours of conjunctions are indicated

Figure 14 left panel shows the position of quasi-simultaneous magnetopause crossings with SMILE, THEMIS A, D and E in YZGSE plane. These represent all crossings during the three years of the SMILE nominal mission. We can see that SMILE will be at the magnetopause with either THEMIS A, D and E for a total of 1173 hours. Out of these SMILE will have about 400 hours of conjunctions with each of THEMIS spacecraft. If we restrict the conjunctions to the ones when SMILE, THEMIS D and E would be at the magnetopause at the same time we still obtain almost 300 h.

Figure 14 right panel shows the position of magnetosheath crossings with SMILE, THEMIS A, D and E in YZGSE plane. Since the magnetostheath is a wider region than the magnetopause there are about 4 times more conjunctions. Similar conjunctions between SMILE and MMS are expected, although with less crossings since MMS has an orbit period around 3.5 days while THEMIS orbit is much shorter around 1.1 day.

It should be noted that during these conjunctions the SMILE SXI instrument will generate an image of the magnetopause every 1-5 minutes and will give the large scale shape and position of the magnetopause that we will compare with in-situ measurements. Furthermore, SMILE will also be taking images of the magnetopause while located in the magnetosheath or magnetosphere (up to 40 hours every 51 hours) and any crossing of the magnetopause by THEMIS or MMS would be crucial to verify the methods to obtain the magnetopause position from the SMILE SXI images.

Finally, when MMS or THEMIS are in the plasmasheet, observing storms and substorms with in-situ plasma measurements, SMILE will be taking pictures of the auroral oval at minute time scale to simultaneously measure the energy deposited in the polar ionosphere.

## Conclusions

The SMILE mission will investigate the Sun-Earth relation in soft X-rays for the first time, using an instrument only used up to now by astronomy missions to study intense X-ray sources in the Universe. The dayside magnetosphere, in particular the magnetosheath and polar cusp, is a source of soft X-ray produced by solar wind charge exchange; highly ionized ions pick-up an electron from the Hydrogen atoms in the Geocorona and subsequently emits an X-ray. Using a large FOV of 15.5° × 26°, the soft X-ray instrument will be able to image the magnetosheath and magnetopause over a large region of space, up to 8 R_E_, perpendicular to the Sun-Earth line. It will also image the polar cusps at mid-altitude.

SMILE will also study large perturbations of the magnetosphere such as substorms and storms using the longest continuous auroral UV imagers. With the high apogee (20 R_E_ geocentric) polar orbit, the auroral oval imaging could be done up to around 45 hours every orbit of 51 hours. Such long observation period will shed new light on geomagnetic storms produced by CMEs impacting the Earth’s magnetosphere.

The ion instrument and magnetometer will measure the incoming solar wind plasma that are driving the changes in the Earth magnetosphere. The ions instrument with two sensors will measure the full 3D distribution function up to every 250 ms, comparable to modern ion instruments such as the ones flying on MMS. The SMILE magnetometer will measure the magnetic field with two identical sensors at different distances from the spacecraft to allow the suppression of the spurious magnetic fields coming from the spacecraft and make very good measurements of the magnetic field in the magnetosphere and solar wind.

The SMILE mission is building upon the collaboration between ESA and CAS that started from the Cluster-Double Star collaboration, while Cluster was lead by ESA and Double Star was lead by China. SMILE, on the other hand, is a 50/50 share between ESA and CAS that started from concept studies and mission selection, followed by joint development and implementation. SMILE is a CAS spacecraft with payload from China and ESA Member States that will be launched by the European launcher Vega-C. It will be jointly operated for a nominal mission of 3 years.
